# Elephant Scar Prevalence in the Kasigau Wildlife Corridor, Kenya: Echoes of Human-Elephant Conflict

**DOI:** 10.3390/ani13040605

**Published:** 2023-02-09

**Authors:** Lynn Von Hagen, Chase A. LaDue, Bruce A. Schulte

**Affiliations:** 1 College of Forestry, Wildlife, and Environment, Auburn University, Auburn, AL 36849, USA; 2 Department of Biology, Western Kentucky University, Bowling Green, KY 42101, USA; 3 Department of Conservation and Science, Oklahoma City Zoo and Botanical Garden, Oklahoma City, OK 73111, USA

**Keywords:** African savanna elephant, conservation, crop-foraging, human–wildlife conflict, human–wildlife interactions, *Loxodonta africana*, social-ecological systems, Tsavo, wildlife management

## Abstract

**Simple Summary:**

African elephants commonly enter farmlands across their ranges and trample or consume crops, creating conflicts with farmers and challenges for conservation priorities. Farmers may retaliate and injure elephants, but the extent to which this occurs is relatively unknown. However, scars found on elephants may be evidence of past negative interactions. The objective of our study was to describe the presence of scars in an elephant population in the Greater Tsavo Ecosystem, a region of high human–elephant conflict in Kenya. We monitored and catalogued a population in the Kasigau Wildlife Corridor from 2017 to 2021, noting the number of scars and their placement on individuals. Presumably, evidence of conflict between elephants is more likely to be found on the head or rump areas of elephants, whereas humans are most likely to injure the body of elephants. We found that adult males were much more likely to have scars than adult females, older males were more likely to have scars than younger males, and more scars were located on the body as opposed to the rump and head. Understanding how many elephants in a population have scars and the location of scars can describe the potential level of human–elephant conflict in a population. This valuable information can be used to better understand the level of conflict in a community and aid in management recommendations.

**Abstract:**

Human–elephant conflict (HEC) compromises crop security and threatens elephant conservation. Most commonly, HEC manifests as crop-foraging as elephants modify natural foraging strategies to incorporate crops. Farmers may retaliate by frightening or harming elephants, leaving scars from inflicted wounds. We assessed the prevalence and distribution of scars on the bodies of African savanna elephants (*Loxodonta africana*) observed in the Kasigau Wildlife Corridor (KWC), part of the Greater Tsavo Ecosystem of Kenya, where conflict is prevalent. We surmised that scars on the body are largely a result of HEC as opposed to scars on the rump or head, which we attributed primarily to elephant–elephant conflict. We hypothesized that: (1) male elephants would have more scars than females; (2) older males would be more likely to have scars than younger males; and (3) most scars would be located on the bodies of elephants. We assessed scars from a photographic catalogue of elephants from the KWC. In line with our hypotheses, male elephants were more likely to have scars than females (32% of males compared to 6% of females); older males had significantly more scars than younger males (61% compared to 24%); and the majority of scars (89%) were located on the body. Scar presence may be useful as an animal-centered indicator to estimate the prevalence and demographic patterns of HEC.

## 1. Introduction

As wild spaces continue to become reduced or fragmented, wildlife and human populations increasingly come into contact [1,2,3]. Negative interactions occur when humans and wildlife compete over resources such as food, water, and space, creating human–wildlife conflict (HWC) [4,5]. Conflict can result in injury or death for people, wildlife, or both, as evidenced in vehicle collisions [6,7], wildlife attacks on people [8,9], or the intentional killing or injury of wildlife deemed a “nuisance” by stakeholders [10,11]. More common than physical harm are the impacts to rural livelihoods in farming communities, as wildlife and farmers clash over food and water resources. HWC is especially impactful in the form of crop-foraging (also known as crop-raiding, when animals leave protected areas to consume crops belonging to local subsistence farmers) in resource-poor communities [12,13,14]. A variety of mitigation methods exist across species and geographies in which crop-foraging occurs [15,16], but evidence of retaliation continues to be prevalent [17,18]. As climate change exacerbates food insecurity [19], instances of injury to wildlife are likely to increase as farmers attempt to protect their livelihoods and families.

African savanna elephants (*Loxodonta africana*) are endangered across their range, with primary threats from illegal hunting for ivory, habitat loss, and negative human–elephant interactions or human–elephant conflict (HEC) [20,21,22,23]. The most common form of HEC is crop-foraging [24,25]; crop-foraging events that compromise an entire season’s harvest in one evening occur, but are rare. However, even infrequent partial losses can impact farmers’ harvests, threatening food security [26,27]. In addition, crop-foraging can create tension between stakeholders, wildlife agencies, and conservation practitioners [28,29]. Given this tension, interventions to mitigate these interactions are crucial. However, the widespread occurrence of such incidents, especially in remote areas, makes mitigation a challenge for management agencies. 

Farming communities in and around the Greater Tsavo Ecosystem (GTE) of Kenya experience high levels of HEC. Elephants are frequent crop-foragers in the GTE, and remain a source of conflict between community members and corresponding wildlife officials [30,31,32]. Between 2001 and 2016 in the GTE, a total of 45,151 cases of HWC were reported, of which 64.3% involved elephants [33]. Farmers often attempt to mitigate crop-foraging by defending their farms with stationary deterrents, active patrolling, or physically frightening away elephants [34,35,36,37]. While most farmers do not attempt to harm elephants around the GTE, a small percentage reported doing so. In a survey of 206 farmers, the vast majority reported being fearful of elephants [38], which could lead to adverse actions by farmers, threatening elephant and human safety or health. Managing and mitigating HEC can also take time and use valuable conservation resources, as wildlife officers sometimes need to perform costly sedations and treatments for injured elephants [31]. 

As a potential reflection of HEC, the presence of conspicuous scars on some elephants is evident in the GTE. Firearms are rarely used in this area due to their illegality and lack of availability, and thus most elephant injuries caused by humans are from arrows or occasionally, spears [33], and most wounds likely go untreated. However, in a study on endangered Asian elephants (*Elephas maximus*) by LaDue et al. [11] in Sri Lanka, gunshots were a common source of injury, especially to male elephants. Obanda et al. [39] used a Kenya Wildlife Service dataset from 1998 to 2007 to evaluate all types of injuries to African elephants in the GTE. Adult males were the most likely to sustain injuries, the legs and body were the most frequently injured body parts, and human-inflicted wounds accounted for two-thirds of injuries. While leg injuries can occur from direct encounters with humans, Obanda et al. surmised that snares and terrain-related causes were the most likely sources of injuries around the legs. Following up on these studies, we sought to determine the relationship between scar presence, age, sex, and body location as a measure of direct negative encounters between people and elephants in the Kasigau Wildlife Corridor (KWC) of Kenya, which is part of the GTE. We focused on scars and not on other forms of injuries such as dislocations, sprains, and swellings [39]. We presumed that scars on the head (or possibly the rump) were more likely to result from interactions between elephants (e.g., mutual sparring, dominance interactions), as elephants mainly spar head-to-head. Conversely, we assumed human-caused scars would be more likely to occur on the body; this area represents a larger target for arrows or spears and other sources of human-inflicted injuries, and humans are unlikely to approach elephants head-on for fear of being detected. Furthermore, we surmised that an elephant would turn away from an imminent anthropogenic threat, leaving very few human-induced scars on the head.

We hypothesized that: (1) adult males were more likely to have scars than adult females; (2) older males were more likely to have scars; and (3) most scars on males would be located on the body as opposed to the head or the rump. Understanding more about how scars are distributed amongst the demographics of an elephant population can give insights into the potential patterns of negative direct human–elephant encounters, which are dangerous to elephants and people. These insights can also assist conservation practitioners in developing management and mitigation strategies. 

## 2. Materials and Methods

### 2.1. Study Area

**Figure 1:**
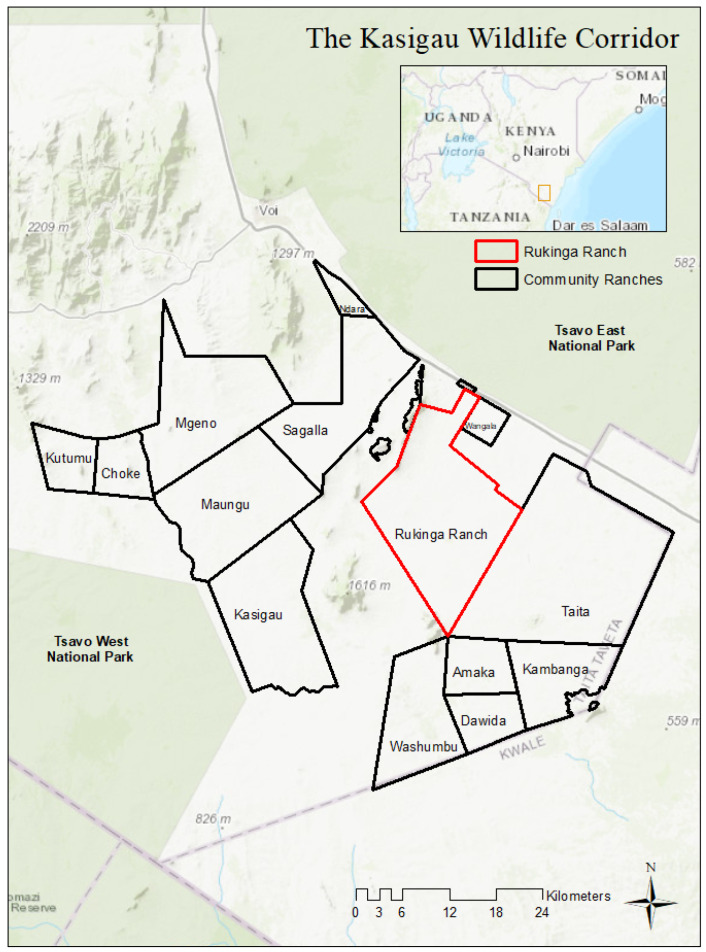
The Kasigau Wildlife Corridor (KWC) of Kenya, showing the location of Rukinga Ranch Wildlife Sanctuary.

The KWC consists of community ranches and lies between Tsavo East and West National Parks in southeastern Kenya (Figure 1) as part of the GTE. The region is home to the country’s largest and growing elephant population of ca. 15,000 individuals [40], and many elephants use the corridor to move between the two parks [41,42]. Rukinga Wildlife Sanctuary, a former cattle ranch and one of the 14 ranches in the corridor that are part of the KWC’s Reducing Emissions from Deforestation and Degradation (REDD+) project, is managed by Wildlife Works. The organization excluded human settlements and livestock grazing to restore the ecosystem function of the ranch, making it a favorable habitat for elephants and other wildlife. In exchange, the organization provides jobs, educational opportunities, and disbursements to local communities, which can be used for numerous improvement projects. The REDD+ project area now contains a subset of the larger elephant population numbering around 2000, with 300 to 500 resident elephants shifting seasonally around the sanctuary [43].

### 2.2. Elephant Observations and Cataloguing

From 2017 to 2019 and in 2021, we monitored and catalogued elephants passing through the sanctuary from approximately May to late January over the two crop-growing seasons. Elephant observations were collected from six different structured wildlife biodiversity driving transects (which covered portions of the 30,000 ha sanctuary), opportunistic sightings, and images from camera traps placed in Sasenyi village for an elephant crop-foraging study [34]. Transects were originally conducted three times per week approaching dusk and starting around 16:45. During the survey periods, we selected one of the six transects randomly without repeating the same transect, until each of the six transects were performed. In 2019, we shifted to one transect per month until natural forage was present, as wildlife presence greatly diminished during drought periods. Information from all transects informed our study. All elephants were recorded and photographed (if possible) when encountered. Specifically, we noted the number of elephants in a group, type of group (family (matriarchal), bachelor, mixed, or lone individual), sex, estimated age and age class, and any special characteristics (such as presence of scars or injury). Approximate elephant ages were determined using visual indicators such as height, height relative to other elephants, wrinkle prevalence, tusk size and length, and head shape [44,45,46]. We subsequently assessed all photographs to confirm field sightings and/or fill in any gaps in data that could not be ascertained from the original sighting (e.g., sex, age) [45,46,47]. Elephant age classes were assigned for males and females (Table 1) [45,47,48]. Prime males are distinguished from young adult and senescing males because prime males are most likely to enter musth, a heightened period of reproductive activity associated with increased aggression and movement [49]. To ensure that the age estimates of adults were accurate from original sightings, each of the authors listed their age group estimates (disagreements occurred in <15% of cases) and those with any differences were resolved through further examination of photos and 100% consensus. For all elephants, a photograph of each ear, their full front, each side, tusks, and rear were added to assist with identification. Because males (especially those aged **≤** 20 years) without distinguishing markings on their ears or variation in tusks often looked similar, we required a minimum of a clear picture of one ear and both tusks to have enough information to re-identify a bull. Ear markings vary between individual elephants as they receive tears and notches from sparring and moving through the bush, and ear vein patterns are also unique. Tusks also have variation in breaks and wear patterns. Thus, we used a combination of ear and tusk markings to identify individuals and noted each recurrence. 

**Figure 2:**
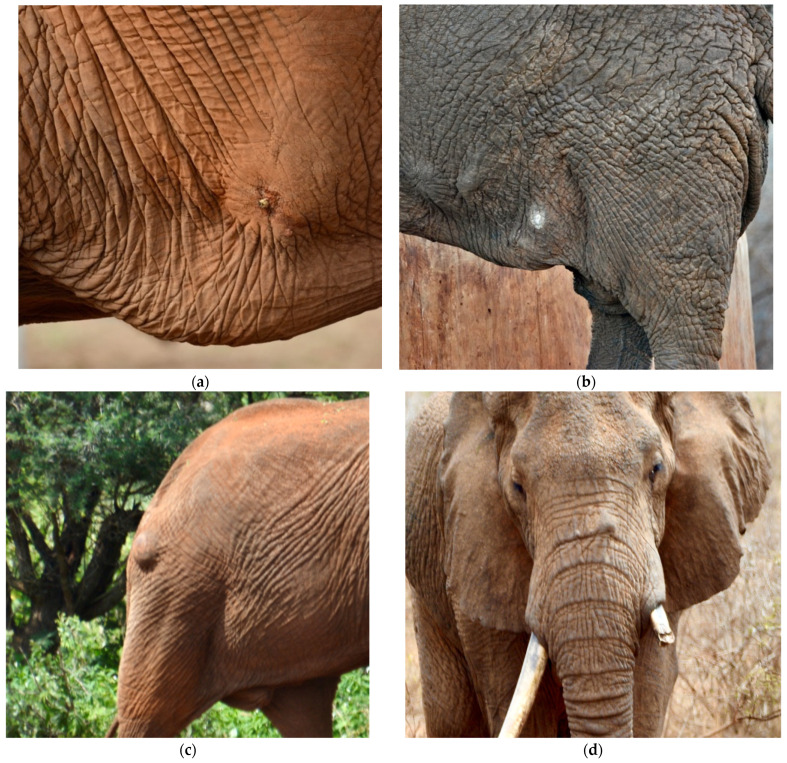
Examples of scars on African elephants. (**a**) A recent injury that is still healing found on the body of a bull; (**b**) a bull with multiple scars on its body; (**c**) a bull with a scar on its rump; (**d**) a bull with a scar on its head. Photos by R.L. Von Hagen.

**Figure 3:**
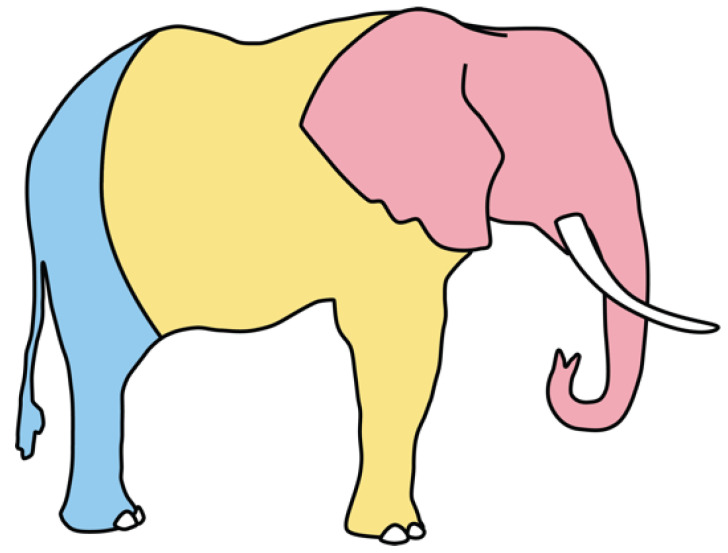
The three body zones of each elephant used to describe the position(s) of scars: head (shown in pink), body including front legs (yellow), and rump including hind legs (blue).

We define “scar” here as any raised area(s) found on an elephant (weeping, scabbed, or healed) that does not appear to be a natural aberration (examples, Figure 2). Whenever scars were noted on an individual, a photograph was taken that included the scar, and its position on the elephant was noted in the catalog. We classified scars into three zones (Figure 3): head, body, and rump. This project operated under the Kenya Wildlife Service’s PIC/MAT agreement with Wildlife Works and with approval from NACOSTI, Kenya’s science agency (License No. NACOSTI/P/20/2292).

### 2.3. Statistical Analysis

For our first hypothesis, we tested for a significant intersexual difference in the presence of scars using Fisher’s exact test (accommodating for small sample sizes) among elephants that we presumed had reached sexual maturity: all males 15 years and older, and adult female elephants 20 years and older [50]. The sample of adult female elephants that had visible scars was very small (*n* = 3). Therefore, we did not continue with any inter-sex analyses. However, the ages of the male elephants included in this study followed a normal distribution (Kolmogorov–Smirnov test: D = 0.068, *p* = 0.319). To examine our second hypothesis, we tested for a significant difference between the mean number of scars between adult males and prime adult males using a Welch’s two-sample *t*-test for unequal variance (we did not have any senescing males in our sample). To examine our third hypothesis, we tested for a significant difference between the presence of scars in the three scar locations (head, body, and rump) amongst all age categories of adult male elephants (age 20 years and older; again, including pubescents did not change the outcome). Because body scars were the most prevalent, we used two separate Fisher’s exact tests: one with head and body and one with rump and body. Alpha values were set at 0.05, and data were analyzed in R [51].

## 3. Results

Males 20 years of age and older were more likely to have scars (*n* = 142/161) compared to adult females (*n* = 3/48) (*p* < 0.001). Furthermore, prime adult males were significantly more likely to have scars than adult males (*p* < 0.001). Of all adult males sampled (≥15 years), 32% had one or more scars, including 25% of adult males (*n* = 24/98) and 61% of prime adults (*n* = 27/44) (Table 2). Furthermore, prime adult males had significantly more scars (*n* = 57; almost twice as many) compared to adult males (*n* = 33; *t*_43.61_ = −2.66, *p* = 0.011). We also found a significantly greater preponderance of scars on the body than either the head or the rump for adult male elephants (both pairwise comparisons, *p* < 0.001): 89% on the body, 4% on the head, and 7% on the rump.

## 4. Discussion

We found clear support for our hypotheses predicting demographic patterns of scars observed on African savanna elephants in the KWC. Specifically, (1) adult male elephants were much more likely to have scars than adult females; (2) older, prime adult male elephants (35–54 years) had significantly more scars on average than adult males (20–34 years); and (3) most of the scars we observed were located on the main bodies of elephants compared to the head or rump. In contrast to a prior study on elephant injuries, rarely were any scars or injuries noted on the legs of elephants [39]. We did not detect scars on males or females below the estimated age of 20 years (sexual maturity in males). While we make reasonable assumptions about the cause of the scars we observed, the use of scar patterns is still informative for wildlife managers as they seek to assess populations’ overall wellbeing and aid in the conservation of this endangered species [39,52,53]. We assume many of the scars that we observed, especially those on the body, were acquired from direct encounters with humans [39,53,54]. Increased tension from ongoing HEC is leading many community members to engage in direct conflict with elephants in the GTE, as has occurred in other elephant ranges where HEC is prevalent [55,56,57,58,59,60]. As such, the prevalence of human-inflicted wounds (and scars resulting from those wounds) may increase until effective HEC mitigation strategies can be more widely implemented. 

From other studies in areas in which African savanna elephants are likely to engage in crop-foraging, males are more likely to be involved in HEC compared to females [61,62,63,64]. Males may be more willing to exhibit risk-prone behaviors (e.g., crop-foraging) to maintain high body conditions while simultaneously traveling between female groups to locate receptive mates [65,66]. Because males had more scars than females in our study, the difference between the sexes also suggests that many of these scars were incurred during crop-foraging, as the sex bias in crop-foraging occurs in the KWC as well [30,67]. Furthermore, scars were most prevalent on the body, compared to the head or the rump, where scars from sparring among conspecifics presumably would be more common [68,69]. 

Given that 32% of male elephants in our study had scars, our study demonstrates that scars may be efficacious as a low-cost method to monitor the prevalence and demographic patterns of HEC involving resident elephant populations in this area, even if a proportion of the scars are from sources other than HEC. This type of monitoring could be in addition to farmer reports, which can sometimes be understandably impacted by bias. A similar method was used by LaDue et al. [53] in Sri Lanka to monitor HEC around a protected area for Asian elephants. In that study, males were also observed to have more scars than females, and males appeared to acquire more scars as they aged. Thus, the authors of that study concluded that gunfire was not an effective deterrent for crop-foraging elephants at their study site, with many older elephants observed with dozens of scars incurred from gunfire. In other words, if scars are reflective of a male elephant’s propensity to engage in conflict with humans, then a direct relationship between scar count and age may indicate that certain deterrents (e.g., gunfire, arrows) are not effective in curbing HEC. As in the Sri Lankan study, we found that, in the KWC, male elephants in the older age class (prime adult, 35–54 years) had more scars than individuals in the younger adult male age class (20–34 years). This indicates that older males may be more likely to have HEC encounters, accumulating scars over time. However, a lack of scars in older males could also indicate that certain individuals are more “successful” crop-foragers, or adept at avoiding farmers, further complicating our knowledge on the source of scars. Furthermore, if the presence of scars is unequally distributed among males within an age group, such a pattern would indicate certain males are more likely to crop-forage. This information is important for developing animal-centered approaches to mitigating HEC.

Guns are not the primary means of injuring elephants in the KWC, but arrows (especially those that are poisoned) can be lethal to elephants; spears, snares, and poisoned pumpkins are all tactics that have also been used in response to crop-foraging [70,71]. As we were unable to ascertain the exact causes of the scars we observed, and because there is a limited understanding of wound healing in elephants [72], future work should describe how often scars are obtained from various sources (i.e., from conspecifics, the physical environment, and negative interactions with humans) and the progression of scarring in wild elephants. This is impetus for further research to obtain a more complete profile of the prevalence of injuries. Such research would help to inform conservation and management strategies for free-ranging elephants and determine the consequences for elephant health. 

While the issue of HEC is complex and should be informed by research from a range of disciplines, there is particular value in adopting an animal-centered approach to understanding some of the drivers of this conflict [73]. We have justified our presumptions on the source of scars in this population with knowledge of elephant behavior. Thus, our study is distinctly useful in this regard, as scars may act as a real-time, adaptive method with which we can monitor HEC with an understanding of elephant behavior and ecology. For example, male elephants regularly undergo a heightened sexual state of “musth”, which makes males more attractive as mates. However, musth requires males to maintain a high enough body condition to sustain this state [74,75]. Thus, males must balance an inherent trade-off: a male elephant may opt to forage so that it can prolong musth, but at the cost of acquiring mating opportunities [49]. Human crops are calorically dense and may act as a readily available, nutritionally rich food source for these males, who in turn may be prone to take risks to acquire these resources. The results of this study seem to support this paradigm, as male elephants were more likely to have scars than females, and older males (who appear to be preferred as mates over younger males [76,77]) had more scars than younger males. However, if scars were more prevalent among females in a population than what we found, then it is possible that management approaches would need to be adapted accordingly. For example, females travel in larger groups than males, and these groups include offspring [78,79]. Additionally, family groups tend to move less per day and stay in one region longer than adult males [80,81,82,83]. Thus, crop-foraging by the same family groups could be an ongoing issue compared to males that may come and go from particular farms. Indeed, two groups of females with some of their calves have already been observed crop-foraging near Rukinga Ranch [84], demonstrating the potential for variation in local populations. While the majority of the communities around the KWC desire to coexist with elephants, many members feel as if local wildlife officials are not able to adequately control crop-foraging [85]. Observing scars can help guide management strategies to conserve endangered African savanna elephants, but ultimately management practices will also need to be informed by a sound understanding of how individual and family groups of elephants behave and navigate human-dominated landscapes.

## 5. Conclusions

Our study described the demographic patterns of scars observed on African savanna elephants in the KWC in southeastern Kenya. Most of the scars we observed were located on the body, and we surmise that these resulted from injuries during negative interactions with humans. Thus, we suggest that these scars reflect trends in HEC. Our study makes reasonable assumptions as to the origins of scars, yet points to the gap in knowledge of how elephants acquire injuries from various sources in the wild. However, our results indicate, as expected, that adult male elephants had more scars than adult females, demonstrating that males are more likely to engage in crop-foraging. During these interactions, humans may directly retaliate with activities such as shooting elephants with arrows or spears in the KWC, and with bullets in other areas. Furthermore, prime males (35–54 years) had more scars than younger adult males (20–34 years), indicating that these retaliatory activities were not necessarily effective in curbing crop-foraging among the male elephants in this area. Interestingly, we found no evidence of scars on males or females in the 10–19 year age class. To validate the methods proposed in this study, there are opportunities for further research to describe how scars occur in elephants, how they heal and persist among elephants, and the threat posed from these injuries to elephant conservation priorities. 

## Figures and Tables

**Table 1:** Age classes and respective ages used to categorize elephant groups.

Sex	Age Class	Age (years)
Male	Calf	<5
	Juvenile	5–9
	Pre-Adult (1A) *	10–14
	Young Adult (1B) *	15–19
	Adult	20–34
	Prime Adult	35–54
	Senescing Adult	≥55
Female	Calf	<5
	Juvenile	5–9
	Pubescent	10–19
	Adult	≥20

* For males, the pubescent category (10–19) was separated into pre-adult and young adult [45]. Young adults have usually left their family group, while pre-adults are still with the family group.

**Table 2:** Scar prevalence among 161 males in the Kasigau Wildlife Corridor of Kenya (see Table 1 for ages).

Age Category	Total Number of Males	Males with Scars	Percent of Sample with Scars	Mean ± SE Scars per Elephant
Young Adult (1B)	19	0	0	0
Adult	98	24	25	1.3 ± 0.09
Prime Adult	44	27	61	2.15 ± 0.21
Totals	161	51	32	1.76 ± 0.10 *

* Young adults excluded from mean ± SE.

## Data Availability

The data presented in this study are available upon reasonable request from the corresponding authors.
